# Approval Delays in Multi-Country COVID-19 Trials: The Case of COPCOV and the Risk of Therapeutic Inertia

**DOI:** 10.1186/s13063-025-09300-z

**Published:** 2025-12-16

**Authors:** Janelle Winters, William Schilling

**Affiliations:** Humanitarian and Conflict Response Institute School of Arts, Languages and Cultures University of Manchester, Ellen Wilkinson Building, M15 6JA, Manchester, United Kingdom; Centre for the History of Science, Technology and Medicine Faculty of History, University of Oxford, 45-47 Banbury Road, OX2 0AD, Oxford, United Kingdom; Mahidol Oxford Tropical Medicine Research Unit, Faculty of Medicine, Mahidol University, Bangkok, Thailand; Centre for Tropical Medicine and Global Health, Nuffield Department of Medicine, University of Oxford, Oxford, United Kingdom

**Keywords:** Multi-country clinical trials, COVID-19, ethics and regulatory approval, global health governance, risk perceptions

## Abstract

**Background:**

Many multi-country COVID-19 clinical trials, including those for widely available repurposed drugs with strong safety profiles, were conceptualised quickly but were unable to influence clinical treatment guidelines. The Chloroquine/Hydroxychloroquine for the Prevention of COVID-19 (COPCOV) trial, a large multi-country clinical trial sponsored by the University of Oxford, sought to determine the efficacy of hydroxychloroquine and chloroquine as a prophylaxis for COVID-19, but faced approval delays and other bureaucratic challenges. Understanding the reasons for these delays will help to guide reform for future multi-country trials responding to health emergencies.

**Methods:**

Using an extensive case study of the COPCOV trial, we aimed to quantitatively and qualitatively analyse the bureaucratic challenges facing academic researchers seeking trial approval across multiple countries during health emergencies. We measured the median time from first COPCOV trial protocol submission to an ethics/regulatory body in each country to first approval and disaggregated the average and median time for approval by ethics committees and regulatory bodies. These data are extracted from official documents in the Trial Master File, records from country investigators, and thousands of stakeholder emails. Additionally, we conducted semi-structured interviews with 65 trial stakeholders to identify barriers to approval and we analysed these interviews using inductive thematic analysis.

**Results:**

For the COPCOV trial, investigators sought approval in 76 countries and submitted initial protocols to 22 local/institutional ethics committees or institutional research boards, 19 multi-site or national ethics committees, and 14 national regulatory authorities. The median time for the study to receive an initial decision (approval or rejection) in each country was 104 days (IQR 42). Approximately half of the countries to which the COPCOV protocol was submitted had sequential systems for ethics and regulatory review, and those with an expedited review system communicated faster decisions (median 91 days vs. 122 days). Issues with efficiency, flexibility, and decision-making coherence underpinned these approval delays. Efficiency challenges included overlap in comments between ethics bodies and duplicative ethics and regulatory body roles. Delays due to inflexibility resulted from under-awareness of existing risk-based frameworks for repurposed drugs, few mechanisms for streamlining documentation requirements during emergency review processes, and under-utilisation of regulatory agility and reliance mechanisms. Objectivity and coherence of decision-making by trial approval bodies was limited by a lack stringent regulatory authority transparency and limited communication channels between trial stakeholders.

**Conclusions:**

Trial approval challenges are rooted in a combination of conservative good clinical practice interpretation and insufficient international guidance and leadership, which contribute to a dangerous ‘risk of therapeutic inertia’ in developing evidence during public health emergencies. Governance reforms to address these challenges should be two-fold, focused on improving national awareness, buy-in, and financing for existing harmonisation and risk-based structures, and establishing a global framework for clinical research during health emergencies.

**Trial registration:**

A separate article reports the results of the COPCOV trial, registered on 11 March 2020 at ClinicalTrials.gov (NCT04303507).

## Background

During the same week that the World Health Organization (WHO) declared COVID-19 a public health emergency of international concern (PHEIC), clinical researchers at the Mahidol Oxford Tropical Medicine Research Unit (MORU) discussed a trial of the repurposed drug chloroquine/hydroxychloroquine (CQ/HCQ) for COVID-19 [1][2][3][4]. With laboratory data suggesting activity in SARS-CoV-2 [5][6], experience conducting clinical and pharmacological studies on CQ for over 30 years, and nothing else available, they determined that CQ was ‘worth a shot’ for COVID-19 prophylaxis. CQ is one of the single drugs to which humans have been most exposed [7, 8], including billions of treatment courses given per year for malaria prophylaxis and treatment since the 1950s and later for rheumatoid arthritis and lupus treatment. It has a strong safety profile at the dosages suggested for COVID-19 prophylaxis [9][10].

The COPCOV trial, a multi-country, double-blind, placebo-controlled, randomised controlled trial (RCT), aimed to assess CQ/HCQ as a prophylaxis for COVID-19. MORU secured COVID-19 Therapeutics Accelerator funding for the COPCOV trial in mid-March 2020 and planned to enrol at least 20,000 healthcare workers (HCWs) worldwide, later increased to 40,000 after HCQ was donated, with a primary endpoint of preventing symptomatic COVID-19 illness [11]. COPCOV’s design aligned with the WHO’s emphasis on ‘the need for large randomised trials with a control group rather than many small and inconclusive studies’. The choice to recruit across multiple countries was based on statistical calculations that assumed that the majority of HCWs taking HCQ as a prophylaxis would not be exposed to COVID-19 (event rate) and that HCQ would have a relatively modest therapeutic impact on preventing disease (effect size) [12]. In addition, it proved difficult early on to predict which countries may be affected. In mid-March 2020, investigators began the first steps for ethics and regulatory approvals in the United Kingdom (UK), Italy, and Thailand [4][13]. COPCOV ultimately became the world’s largest pre-exposure prophylaxis trial for COVID-19 [3]. Yet, despite obtaining the ‘golden ticket’ of UK National Institute for Health Research (NIHR) Urgent Public Health designation in March 2020, which positioned it as one of a small group of trials to be prioritised for National Health System resources [14], the trial recruited less than 5,000 participants worldwide and published its results four years after the pandemic began [15].

The COPCOV trial missed the opportunity to recruit during peak COVID-19 caseloads and to contribute to the evidence base for antiviral prophylaxis when this would have been most valuable. Its focus on HCQ subjected COPCOV to more political forces than most COVID-19 clinical trials. Yet, the COPCOV experience reflects an approval and recruitment situation that is not new. During the H1N1 influenza response in 2009 clinicians described an ‘epidemic curve of ambition’ characterised by “lots of bright ideas, a few protocols, small sample sizes and zero evidence” [16]. Sigfrid et al. documented at least fourteen studies or committees from 2009-2017 calling for expedited approval processes for ECs during emergencies. Table 1 [17–29] provides a snapshot of pre-COVID committees, projects, and studies that explored the challenges faced by ECs during health emergencies [30]. It showcases how ‘experience from previous epidemics highlights how time and again, the research response is delayed and the narrow window of opportunity for enrolling patients during peak epidemic waves is missed’ [26]. While the epidemic curve of ambition may have particularly damaging health consequences during epidemics, multi-country clinical trial approvals often also proceed at a sluggish pace during ‘peacetime’. There has historically been attrition at each step of clinical research implementation; trial recruitment often misses peak caseloads during epidemics and very few trials publish results with adequate power to guide timely treatment recommendations [31][32].

No known studies have explored the specific EC/institutional research board (IRB) and national drug regulatory authority (NDRA) challenges faced by multi-country trials operating across multiple regions during COVID-19. The few studies that have quantified clinical trial approval experiences during health emergencies were limited to a particular EC body [20, 23, 33] or trial operating within a single region. For instance, researchers documented review times and approval challenges for COVID-19 trials in the European Union, including the DisCoVeRy, REMAP-CAP, and multiple arms of the SolidAct trial. These demonstrated a wide variation in approval times across European sites and countries, ranging from seven days to over 12 months, with multi-country trial dossiers requiring up to 535 documents [14, 34, 35].

In this context, we consider the forces that continue drive multi-country clinical trial attenuation. Based on a quantitative and qualitative analysis of the COPCOV trial’s approval experiences, we argue that protracted approvals delays were related to challenges with flexibility, efficiency, and coherence of decision-making processes. As evidenced by Table 1, these challenges were anticipatable and remediable. They are the product of risk-averse bureaucratic structures that overlook the risk of scientific attrition i.e., the loss of potential life-saving scientific benefits. Their persistence has profound implications for access to potentially life-saving, affordable medicines in resource-limited settings, and if not addressed will likely recur in future pandemics.

## Methods

The aim of this research is to quantitatively and qualitatively analyse the challenges facing academic researchers seeking trial approval across multiple countries during health emergencies. Our analysis is based on extensive primary document analysis and semi-structured interviews. Quantitative data analysed included time from first submission to ethics/regulatory authorities (EC/NDRA) to approval or denial of permission to recruit in each country and aggregate time for approval across countries. These descriptive data were compiled from documents in the COPCOV Trial Master File and thousands of emails sent between site investigators and the MORU research team from March 2020 to January 2022. They were supplemented by semi-structured interviews with 65 trial stakeholders, conducted from April 2022 to September 2023. Interviews, summarised in Additional File 1, were conducted with permission from the University of Oxford’s central research ethics committee (CUREC R81146/RE001). Investigators from countries that began any regulatory or ethics committee submissions were invited to interview, regardless of the completion or outcomes of these submissions. Interviews were also conducted with broader trial site teams in four countries (the United Kingdom, Thailand, Mali, and Indonesia) to capture experiences across sites with a broad range of recruitment numbers and geographies. For those that accepted, interviews were semi-structured and aligned with the COREQ checklist (Additional File 2 includes the customisable interview guide), with a focus on emergency ethics or regulatory mechanisms in place; comments received by ECs and NRAs; the presence of sequential or parallel submission requirements; and practices associated with the speed of the approvals process at each site. Transcripts were reflexively, inductively, and manually coded in NVivo 14, using Braun and Clark’s six-stage thematic analysis framework [36].

## Results

### Quantitative

**Fig. 1:**
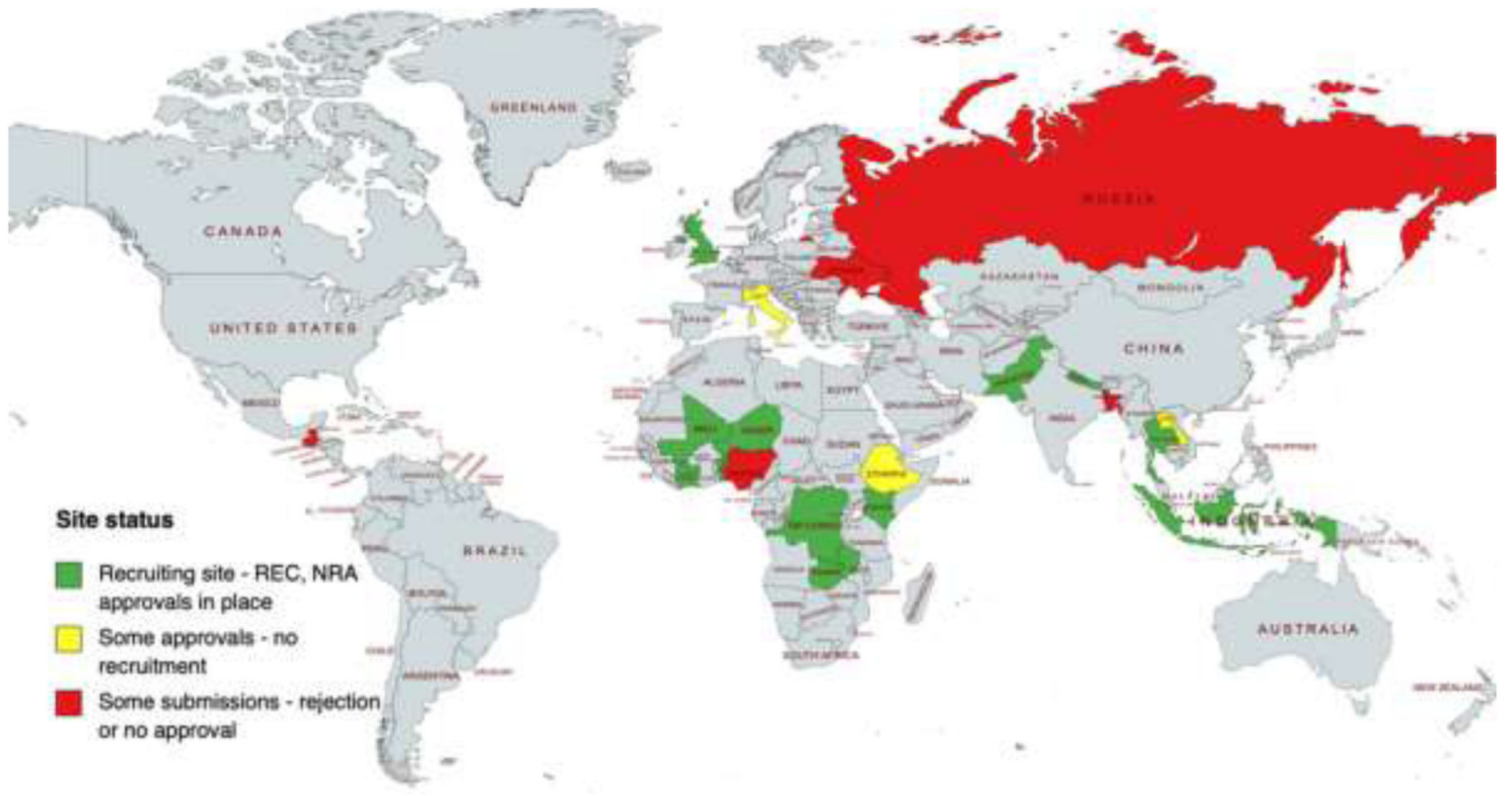
Countries with COPCOV CTA submissions to ethics or regulatory bodies

COPCOV investigators contacted potential site investigators in 76 countries and 11 of these recruited participants (26 sites). The COPCOV protocol was submitted to 22 local or institutional ECs/IRBs; 19 multi-site, regional, or national ECs; and 14 NDRAs for approval (Figure 1). Two different sets of submissions were undertaken for most sites. The first was initial approval for the protocol to recruit HCWs (generally involving multiple versions of this protocol) and the second was for an amended protocol to expand recruitment to community-based participants in early 2021. This amendment reflected the fact that HCWs had already contracted COVID-19 or received vaccines at many sites and were therefore ineligible for COPCOV. Despite initial submissions in seven countries by early May 2020, only two countries (the UK and Thailand) were approved in time to recruit during the earliest 2020 waves of COVID-19 infections. Recruitment was severely impacted by a paper published by Mehra et al. in *The Lancet* in late May 2020 [37]. While the paper – which linked HCQ treatment to cardiotoxicity – was retracted in early June 2020 when it became evident that the authors had analysed a dataset with falsified data [38, 39], it resulted in a month-long *de facto* shut-down by the UK’s NDRA (the MHRA, see Additional File 5). Sites in both the UK and Thailand were unable to recover recruitment momentum after this shut-down was lifted, and NDRAs and ECs in more than half of the countries to which a COPCOV clinical trial agreement (CTA) was submitted for approval continued to cite safety concerns related to Mehra et al. over the next two years. Subsequent delays meant that only these two countries had activated sites with recruitment when vaccines were rolled-out to HCWs in many countries during the first months of 2021.

**Fig. 2:**
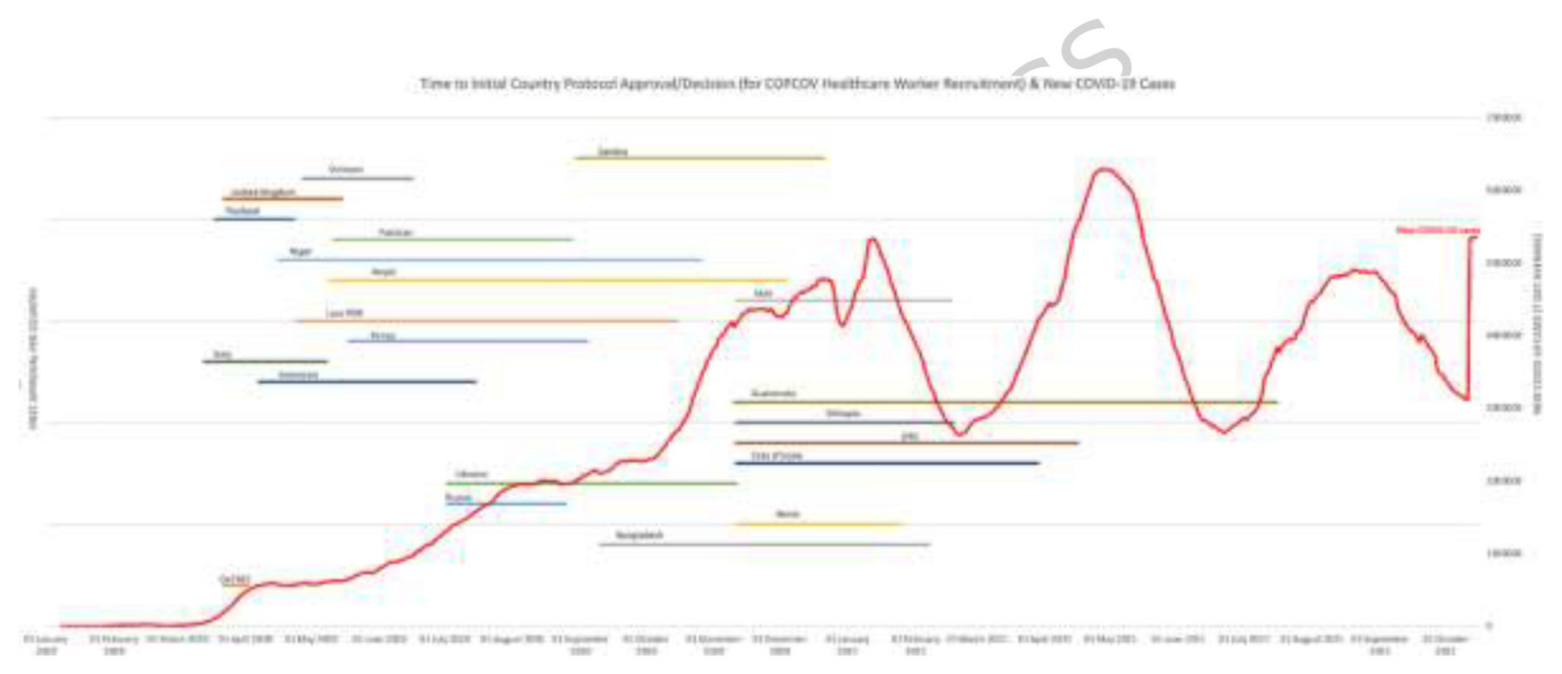
COPCOV time to decision (approval or denial) for HCWs in each country, compared to new COVID-19 cases

**Fig. 3:**
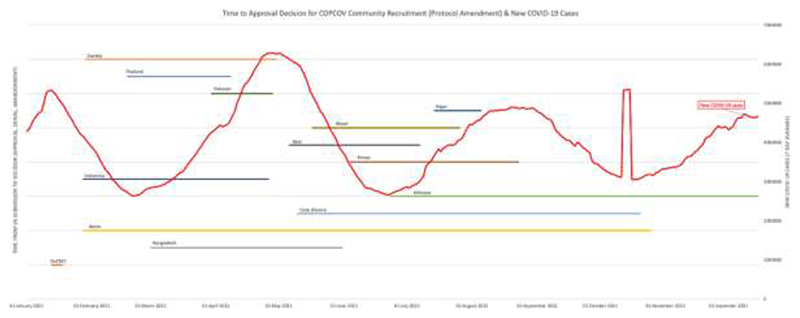
COPCOV time to decision (approval or denial) for community recruitment in each country, compared to new COVID-19 cases

**Fig. 4:**
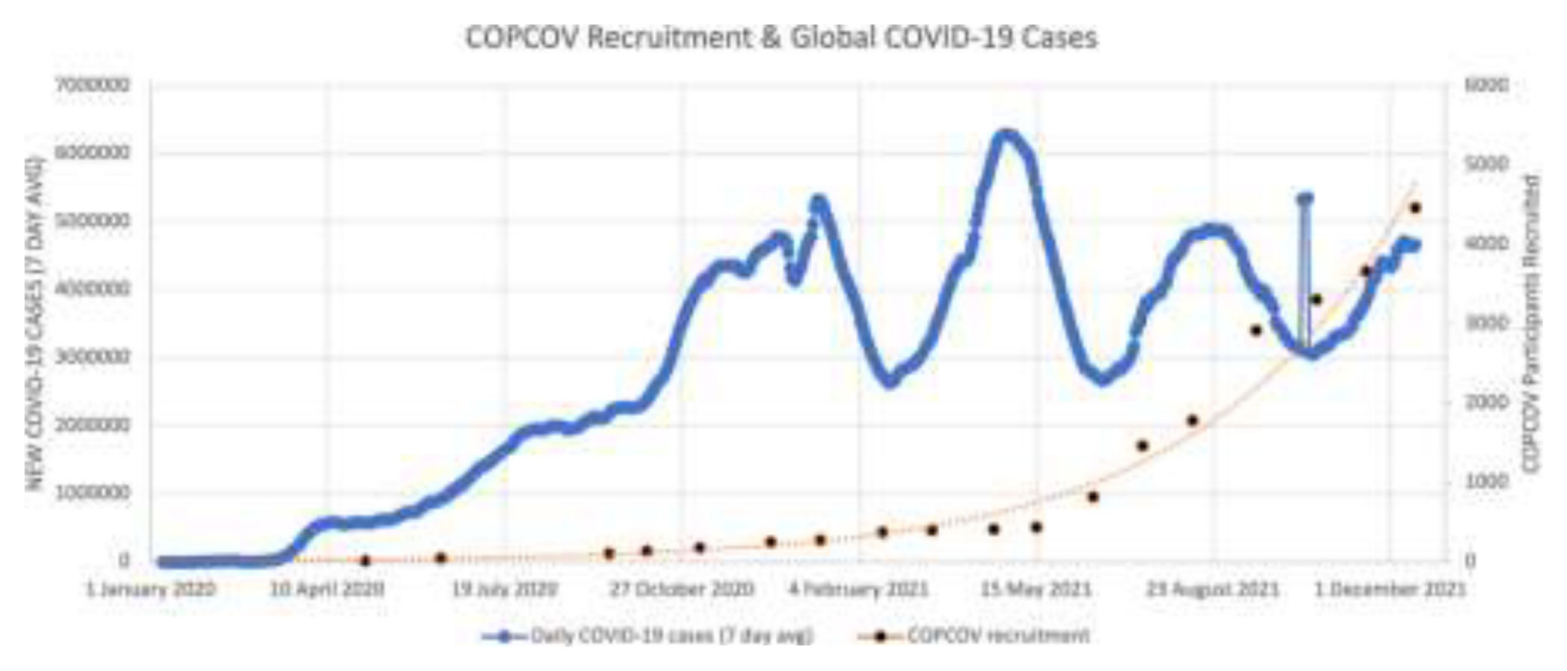
COPCOV recruitment across all sites, compared to new COVID-19 cases

Figures 2 and 3 display each country’s wait time for permission to begin recruiting, and Table 2 shows the aggregated days required for EC decisions and median time for the study to start across countries. As demonstrated by these figures and Figure 4, which shows total recruitment as compared to new COVID-19 cases worldwide reported to the WHO, approval delays contributed to the trial missing early waves. These timelines do not include two major upstream impacts on time to first site recruitment: producing and signing CTAs and procuring clinical trial insurance. For example, a CTA with an Indonesian institution was under consideration for more than eight months due to challenges of negotiating tripartite agreements (between a hospital, university, and the sponsor), budget negotiations, and safety concerns resulting from Mehra et al. that led to requirements for participant screening with electrocardiograms (ECGs, Interviews 13-15). It also took more than a year to finalise the trial insurance policy in Nepal. Additional downstream issues, particularly issuing import permits and handling expiring HCQ/CQ (the investigative medical products or IMP, which became a cascading problem due to delays with approvals) further contributed to delays from national approval to first recruitment.

Approximately half of the countries to which a COPCOV protocol was submitted had sequential systems for ECs and NDRAs. Thirteen of 20 countries with submissions had a formal or informal expedited or emergency review system in place for ethical or regulatory approval COVID-19 studies at the local and/or national level. Countries with some form of expedited system took a median of 91 days for the initial protocol decision (average 95 days), compared to a median of 122 days for those without an expedited system (average 130 days).

**Fig. 5:**
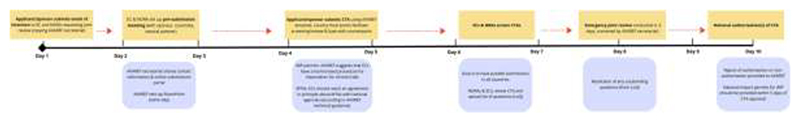
AVAREF’s 10-day emergency review process’s formal timeline, as advertised by AFRO in 2020-2022

When interpreting these figures, it is helpful to consider how ‘timelines all depend on who is counting and when they start the clock, so they’re very relative’ (Interview 44). COPCOV’s experience with the only functioning system for regional harmonisation of clinical trial approvals during COVID-19, the WHO Africa region (AFRO)-based African Vaccines Regulatory Forum (AVAREF), provides an example of how official timelines can obscure lengthy delays. AVAREF countries endorsed a 10-working day goal for ethics and regulatory clearance of trials during health emergencies. Yet, as Figure 5 underscores, approval took a minimum of 50 calendar days for COPCOV and could be seen to take a minimum of 99 calendar days for a decision (164 days to begin recruitment).

### Qualitative

Various lenses can be used to analyse the effectiveness and resilience of national approval systems during health emergencies. Gumber et al.’s systematic review of international clinical trial challenges broadly identified ‘operational complexities’ as major impediments to trial set-up, particularly non-harmonised regulatory approvals and sponsorship structures [40]; Crosby et al.’s retrospective cross-sectional survey of trial site initiations found statistically significant associations between both ‘triage processes’ and mutual acceptance ethics approval mechanisms and the time to site approval/activation [41]; and Glasziou et al.’s governance review of procedural options for research ethics recommended efficiency improvements in the ‘4Rs’ (reducing, reusing, recycling, and replacing) [42]. However, none of these studies were specific to health emergencies and, in the context of the pandemic, sponsors were primarily concerned with two areas: the speed of approval (or rejection) and resources required. In this context, principles of strategic decision-making and organizational theory in the business environment provide a helpful additional lens, including a focus on strategic interdependencies, organizational flexibility, and dynamism. We categorised COPCOV delays into three broad themes: efficiency, flexibility, and decision-making process coherence (Additional File 3).

***Efficiency*** in trial approvals requires building resilience through careful reinforcement of certain types of redundancy within bureaucratic systems (sometimes called strategic interdependencies) [43]. It is a delicate balancing act between building resilience by promoting redundancy in crucial roles, such as having extra staff members to onboard during an emergency, while minimising redundancy in procedural steps, such as long or ambiguous approval chains. Efficient trial approval systems have clearly delineated roles for actors (strategic redundancy), straightforward submission options, and clear communication channels for EC/NDRA queries and decisions. Most COPCOV approval delays were related to deficiencies in at least one of these areas.

All but two trial countries had a NDRA and most had more than one EC. There was considerable overlap in the comments provided by each of these bodies, and sequential approval practices were one of the largest contributors to trial approval delays. EC and NDRA hierarchies are bolstered by the idea that they support local involvement in decision-making about what risks are acceptable for trial participants [44][45]. Additional time for local ethical review is justified by the population-specific perspective that these bodies may have when judging benefits to collective society and individual beneficence [46]. Despite this, the safety risks related to the (fraudulent) [39] findings of the Mehra et al. paper in the *Lancet* were by far the commonest comments, and very few EC/IRBs at a local or national level raised any population specific points. Regulatory authorities in Bangladesh, Ethiopia, Kenya, Nepal, Thailand, and the UK specifically mentioned Mehra et al. in their formal comments on the COPCOV CTA after the article’s retraction and asked for justification of drug safety. As a lead investigator at a prospective COPCOV site explained, professionalisation pressures on ECs in low-resource settings over the past decade may unintentionally contribute to overlap and inefficiency in comments rather than providing critical local perspectives:

… COPCOV shows how everything is getting more and more conservative. Yes, there’s huge pressure on low- and middle-income countries to professionalise and make the ethics committee better. So, ethics committees end up getting tonnes of training and then they feel they have to adhere to the standards… they don’t understand the discretion used in how these are exercised in high income countries. And so it’s like a monster has been created… (Interview 34)

Similarly, numerous site investigators pointed to mismatched expertise at NDRAs for evaluating the safety of clinical trials. They described how NDRA training and staffing in the past few decades has focused on meeting the requirements for new drug licensure, such that investigators needed to explain the RCT approach itself to regulators who lacked a research background (Interviews 9, 55, 61, 65).

Challenges with duplicative EC and NDRA roles were compounded by untransparent submission systems maladapted to the pandemic context. Put simply, electronic submission and tracking systems often existed only on paper. Despite AVAREF’s virtual submission platform, numerous countries required investigators to submit hard copies of documents (Interview 65). Ukraine, Russia, and Vietnam’s strict regulatory infrastructures required notarised documents to be stamped in-person and shipped, which sometimes delayed submissions by weeks. When a lack of transparency of submission requirements was combined with poor direct communication channels between the sponsor and NDRA, there were frequent misunderstandings about EC queries and approval delays (as an example, it took a month for Russia’s rejection to be communicated to investigators). Investigators’ networks and unofficial channels were required in most countries to push-through approvals. Local investigators reflected that ‘some champions can really help sway’ the national EC at an African site (Interview 9), while ‘many diplomatic calls were required to keep the documents moving’ at an Asian site’s EC (Interview 57).

***Flexibility*** within the approvals process is directly related to stakeholders’ perceptions of relative risk (i.e., the likelihood of harm and severity of this harm if it occurs) during a pandemic compared to a steady state. From one vantage, the risk increases because the unknown aspects of the pandemic mean that the trial’s intervention could expose participants to severe harm [47], such that heightened system requirements are instated [48]. From a second vantage, the risk of implementing interventions is lowered because of the potential for the pandemic to worsen and cause severe harm to participants without any efficacious interventions (the trigger for expedited system processes). The international community has ostensibly accepted the latter approach to risk during health emergencies, with the Organisation for Economic Co-operation and Development (OECD) and WHO releasing guidelines recommending reduced submission requirements for repurposed drug approvals [49][50]. There are three major pathways that countries can use alone or in combination to respond to pandemic risks and streamline approval requirements. Countries can track specific trials, such as those for repurposed drugs, on a lower-risk pathway with reduced approval requirements; use expedited approval mechanisms at the IRB/EC, NDRA, or both levels to prioritise pandemic-related trials without necessarily decreasing approval requirements; or apply regional regulatory reliance mechanisms to promote EC/NDRA work-sharing between and within countries.

During COPCOV, most countries approached risk from the first vantage, adding *additional* procedural steps to trial review, such as additional NDRA or independent local EC approval requirements (Interviews 62, 65). No ECs or NDRAs explicitly used existing risk-based frameworks for repurposed drugs to track COPCOV for streamlined approvals. CQ is perhaps the most classic example of a repurposed drug in existence, as it is one of the most widely used drugs in the world, purchasable in nearly all tropical countries (it was previously even added to table salt), and well-known by clinical practitioners [51]. As COPCOV site investigators in Southeast Asia and Africa explained, ‘It wasn’t like there was a new drug, but I think ethics committees tend to behave in the same way regardless’ (Interview 34), and ‘the challenge is that documents are often “required” based on individual misconceptions about risk and what’s mandatory’ (Interview 37). Most study sites were similarly not aware of the WHO guidelines for rapid review of research during public health emergencies (Interview 42) [50].

Emergency expedited review mechanisms were not universal at the national or local level, but they did help curtail delays, especially in countries with sequential approval systems. Across COPCOV countries, expedited review was accomplished through more frequent meetings or streamlined hierarchies (such as designating a single national EC in Italy, having more frequent EC meetings in Thailand, and applying pressure through AVAREF to avoid extra EC requirements in certain countries). It was *not* accomplished by reducing documentary requirements. As White et al. reflected upon over fifteen years ago, sometimes over a kilogram of paper documentation is required for a trial approval, which raises its own set of ethical concerns [52]. Additional File 4 shows how at least 63 discrete documents were required by a single country’s NDRA for COPCOV’s approval. As Additional File 5 shows, streamlined approval processes – such as through the UK’s Integrated Research Application System that combined EC and NDRA approval submissions – led to dramatically fewer document requirements.

Regulatory agility and reliance mechanisms, which are sometimes referred to as work-sharing or mutual recognition, are based on the reflection that communities affected by a pandemic ‘generally have a greater risk tolerance and are more open to medical interventions based on fewer clinical data’ [53]. They have typically been used to allow healthcare providers to prescribe, and medical product manufacturers to provide, countermeasures based on limited efficacy data. For instance, the United States Food and Drug Administration (FDA)’s Emergency Use Authorization (EUA) was used to authorise Tamiflu during H1N1 in 2009, the European Commission’s Article 58 of Regulation 726.2004 for the RTS,s/AS01 malaria vaccine in African countries, and the WHO’s Emergency Use Listing Procedure for Zika in-vitro diagnostic tests [54]. While the WHO has ostensibly supported regulatory agility and reliance in most of its regions, regulatory reliance system functionality has not extended to clinical trial review beyond the Africa region (Interviews 2-4) [55].

Indeed, despite being the only regional platform available to assist COPCOV investigators with harmonisation, no COPCOV countries met AVAREF’s 10-day expedited approval timeline (Figure 5). Most approvals extended not days but months beyond this timeline. AVAREF and the WHO African secretariat have no ability to compel countries that have signed-on to its framework to adhere to the timeline or its embedded system of parallel EC/NDRA review. As a representative at the AVAREF secretariat explained, hard work has gone into obtaining buy-in for the expedited joint review system, but ECs and NDRAs remain ‘fiercely independent’ and often do not allow representatives to make decisions during this joint review (Interview 2). This leads to comments flowing through unofficial channels (Interviews 9, 57).

***Objectivity and coherence*** of decision-making is fostered when ECs/NDRAs operate within their nationally agreed mandates, critically appraise evidence with consistency, and communicate rationales clearly to sponsors [26]. While inflexibility and inefficiency played a major role in delaying approvals in most countries, decision coherence issues were the major drivers of rejection (or having no decision communicated to the sponsors, interpreted as implicit rejection).

NDRAs are designed to vet safety data from preclinical studies, and researchers have cautioned against them becoming a ‘research court’ or conditioning their approvals on efficacy data [45][56]. However, the Mehra et al. paper and the polarizing politics around HCQ [57][58] influenced decisions about COPCOV and led to demands of efficacy data to counteract purported safety issues of CQ/HCQ, with some individuals holding outsized sway on decisions. In an Asian country, for instance, an investigator explained that the national EC was ‘a very good thing…necessary and it gives what we do credibility’ – but that it was simultaneously ‘where major connections count’, and ‘diplomatic cajoling’ of a senior physician running the EC (who was heavily influenced by the politics around HCQ) was needed (Interview 61). A single stakeholder also derailed authorisation at another prospective Asian trial site (in this case, an influential person with responsibility for vaccine coverage indicators, Interviews 14 and 17).

During COPCOV, some stringent regulatory authorities (SRAs) abdicated their responsibility to model objectivity in decision-making. Despite COPCOV’s independent Data Safety Monitoring Board’s unanimous agreement that the trial should continue without any pause [59], the UK’s MHRA suspended all trials with HCQ arms, with one exception. The RECOVERY trial, which used a higher HCQ dosage than COPCOV, was allowed to continue its HCQ treatment arm. The MHRA did not give other trials the green light to re-start for three weeks after the paper’s retraction; COPCOV first heard about the lifted suspension through the MHRA’s website, rather than formal communication channels (Interview 5); and a consultant cardiologist continued to raise questions about CQ/HCQ safety and efficacy for months after the trial re-started. Because the MHRA is a SRA, its protracted pause of the trial diffused safety and efficacy concerns across many less-resourced ECs and NDRAs, delaying evidence about whether HCQ/CQ should be recommended as a prophylaxis in countries that continued to have vaccine coverage in single digit percentages throughout 2021-2022 (while guidelines in about a third of the world continued to recommend CQ/HCQ for COVID-19 treatment or prophylaxis). At least fifteen ECs or NDRAs raised safety concerns that either directly cited Mehra et al. or asked for ECGs to address purported cardiotoxicity (QT interval elongation). Laos and Vietnam authorities – where the COPCOV trial did not move forward – specifically cited the MHRA’s concerns about CQ/HCQ. Later, the MHRA repeatedly questioned investigators about the trial’s continuation given vaccines were already available in the UK.

For some countries, investigators simply never knew whether delays and rejections were related to challenges with efficiency, flexibility, or decision-making processes. A ‘black box’ of unofficial communication channels and poor transparency from ECs and NDRAs meant that investigators never knew why some regulators rejected COPCOV. In Guatemala, for example, national EC approval was required before NDRA approval, but the Ministry of Health became involved in the ethics approval process and shut it down for undisclosed reasons (Interview 62). In Malaysia, untransparent communication channels meant the precise combination of politics, vaccines, and negative press around HCQ that led to the study’s rejection by the Ministry of Health was never known [60][61], even though trials for other politicised drugs including ivermectin were approved [62]. Russian authorities provided a rationale for rejection that cited safety concerns with HCQ, although the government retained the drug on its list of drugs to treat COVID-19 until months later [63][64]. Ukrainian authorities first sent numerous comments to COPCOV investigators to guide re-submission and then rejected the trial outright the next month, citing a blanket order to deny authorisation to all clinical trials for COVID-19 medicinal products. These experiences reflect how transparency challenges remain universal at NDRAs. In the UK, for instance, the 1968 Medicines Act reinforced secrecy of UK regulatory agency approvals [65]. When we submitted a freedom of information request to the MHRA to better understand its rationale for actively continuing the suspension of HCQ trials in 2020, we received heavily redacted committee meeting minutes (Additional File 6), with the explanation that information concerning expert committee members are ‘not in the public domain’ [66]. We were further informed that additional documents are ‘exempt under Section 12 of the Freedom of Information Act’, which ‘allows public authorities to refuse requests where the cost of dealing with them would exceed the appropriate limit’ [67].

## Discussion

Not all health emergencies are best served through multi-country clinical trials but international collaborative trials can be critical for reacting to unknown global transmission patterns and ensuring sufficient recruitment numbers [40], especially for outbreaks with relatively low attack rates. Overall, approvals for COPCOV took too long to provide urgently needed evidence in an unprecedented global medical emergency. This evidence would have been most useful before the arrival of vaccines and thus missed the opportunity to inform therapeutic guidelines in the two years it took most of the world to be vaccinated. The first COVID-19 joint review conducted by AVAREF, for the Drugs for Neglected Diseases initiative’s ANTICOV trial (which also focused on widely available repurposed drugs), faced similar challenges and did not meet the agreed-upon timeline for any country [68]. Even the RECOVERY trial would likely have taken two to three months to launch and missed the spring-summer 2020 waves (critically not developing evidence for life-saving dexamethasone efficacy in COVID-19 treatment) if it had not obtained expedited approval in the UK during March 2020 [16]. Ethics and regulatory processes that contribute to delays while not necessarily reducing patient safety risks – like sequential reviews and cash-before-cover insurance policy papers – have generally increased in recent years. Conversely, transparency about decision-making has not increased. These trends reflect the global proliferation of IRB/EC and NDRA bodies outpacing harmonisation structures and communication mechanisms for multi-country clinical trials [69]. ECs often have wide variabilities in skills, membership, and efficiency [20] and NDRAs have nearly universal resource constraints in Africa for example [70].

Our case study of COPCOV approval bureaucracies has some limitations, which are summarised in Table 3. Quantitatively, by not ‘stopping the clock’ while protocols were being revised, we may over-estimate some approval delays. Yet, we make the case that we comprehensively *underestimate* overall approval times because they do not include upstream delays (e.g. uncertain submission requirements, delays to submit due to missing documents like local insurance policy copies) or downstream delays (e.g. issuing formal import permits for the IMP after NDRA approval). Our stakeholder interviews also have some areas of potential confirmation, selection, and sampling bias. For instance, while all trial sites were invited to participate in interviews, we were able to visit trial sites in-person in Indonesia and Thailand and therefore conducted interviews with a broader number of stakeholders in these countries. Although the interview guide included questions about what went well during the trial, trials were semi-structured and most interviews also delved more deeply into the site-specific challenges faced. We used broad and overlapping categories (flexibility, efficiency, and speed) for our thematic analysis to reduce researcher subjectivity and cite the country/position of interviewees as much as possible to avoid over-generalisation of results. Additional case studies of multi-country trials with other IMPs and countries will be important to determine the broader applicability of our thematic categories.

Numerous ‘common sense’ approaches suggested before COVID-19 [24][42] could have helped streamline approvals for multi-country trials like COPCOV had they been more broadly implemented. These include creating hibernating trial protocols, pre-approving emergency protocols or clinical trial agreement templates, implementing mutual recognition agreements, rolling-out administrative support platforms, and paying EC members for their time. Some potentially promising international initiatives have since been advanced for clinical trial governance reform following the COVID-19 pandemic. The World Health Assembly’s Resolution 75.8 led to a public consultation on clinical trial governance and the first WHO Global Clinical Trials Forum in late 2023 [71]; the Good Clinical Trials Collaborative has published guidance with ‘underpinning principles’ for good (reliably informative, ethical, and efficient) RCTs [72]; the G7’s 100 Days R&D initiative has called for baseline funding for countries and SRA support for streamlining, harmonising, and simplifying regulatory processes [73], with the new 10 Year Health Plan for England aiming to reduce clinical trial set-up time to 150 days by March 2026 [74]; the EMA released a report on European Union actions to improve clinical trial set-up during health emergencies [75]; and the International Council on Harmonization (ICH) is undergoing a revision (R3) of its Good Clinical Practice (GCP) guidelines [76]. Experts from the Global Clinical Trials Forum also recently published a roadmap for improving the timeliness of international clinical trial approvals [77].

Three of the roadmap’s recommendations would have particularly helped with COPCOV approvals. First, EC reliance infrastructures should be strengthened, particularly for trials like COPCOV that use repurposed drugs or IMPs categorised as lower risk (e.g., the OECD framework’s ‘usual care’ and ‘modified use’ risk categories) [49]. Duplicate country EC review processes resulted in few locally specific queries during COPCOV and similar trends have been found for other trials (e.g., DisCoVeRY) [35]. Advancing a single EC model per country or regional economic community for multi-country trials could have saved at least 1,722 aggregate days spent on local EC/IRB approval during COPCOV. Amuasi recently underscored that ethics body harmonisation and capacity-building generally lags behind NDRAs [78]. As Tusino and Furfaro reinforce, such harmonisation must balance expedited ethical review against patient safety risks and incorporate community values, such as by including representatives from all countries and using region-specific templates [56].

Second, parallel and ideally joint CTA review by ECs and NDRAs should be a norm for high-priority multi-country trials. The COPOV case study highlights how regional harmonisation and risk-based structures to support NDRAs and ECs already exist on paper in many regions, but in-country stakeholders are often either unaware of them or perceive them to have limited operationality. To-date, funding has not been forthcoming for capacity building and planning for these approaches; a recent report found that, despite recent pledges from the Bill & Melinda Gates Foundation and some European governments, funding levels for regulatory capacity development are insufficient to meet needs identified by the Global Benchmarking Tool [79]. Initiatives like the European and Developing Countries Clinical Trial Partnership (EDCTP) currently provide grants for NDRA and EC capacity building specific to clinical trials, but multiple COPCOV country stakeholders confirmed that the demand vastly exceeds supply. Platforms like AVAREF require improved national awareness, buy-in, and financing for capacity building to give them ‘teeth’.

Third, it is critical for a specific multilateral actor, ideally the WHO, to take leadership in mapping established processes and legislation for ECs and NDRAs and identifying regional harmonisation gaps. At present, the WHO’s Global Benchmarking Tool for NDRAs and pilot EC benchmarking tool are not universally used and their results are usually confidential [80][81]. Improved transparency would help with requests from countries (as part of the World Health Assembly 75.8 public consultation) for mapping gaps within the global clinical trial ecosystem. Cavaleri et al.’s recommendation of a public database compiling the documents required for NDRA and EC approvals per country [77] would have been especially helpful for COPCOV investigators as they navigated the dozens of ICH-GCP and nationally required documents required across multiple sites. As a technical organisation with the PHEIC infrastructure, the WHO is uniquely fit for purpose in terms of leading and defining special circumstances during which a risk-based approach to clinical trials is triggered (which recognises the high risks of delay and overrides regional frameworks). The recently negotiated WHO Pandemic Agreement makes the helpful recommendation of rapid and transparent publication of trial protocols, which can streamline upstream CTA steps [82]. However, they do not directly address the inefficiency of EC/NDRA approval systems that resulted in the initial COPCOV protocol being considered for 3,886 aggregate days across countries. The WHO will ideally go further in supporting combined EC/NDRA review during PHEICs, with protections for national sovereignty (such as allowing local ECs to have a short opt-out period during which they would provide public reasons for their decision). To be effective, such a multilateral structure would ideally also include centralised mechanisms to support other bottlenecks experienced by multi-country trials, including trial insurance, IMP import permits and shipping, data sharing agreements, and material transfer agreements.

We argue that the burdensome documentary requirements experienced by COPCOV are a product of sponsors and national authorities balancing trial risks and benefits based on narrow definitions of risk. Petryna has described the incorporation of the International Council on Harmonisation of Technical Requirements for Pharmaceuticals for Human Use (ICH)-GCP compliance across academic institutions as a form of ‘bureaucratic formalism’. This term encompasses the interpretation and mitigation of risks based on ‘document-based accountability’ mechanisms [83]. Organisations like MORU have created clinical trial units to meet demands of ECs/NDRAs, which often conform to the rigid risk interpretations of industry. These rules reflect sponsors’ and regulatory bodies’ conceptualisation of risk, which generally focus on reputation and finance. During COPCOV, another major risk was largely overlooked: what we call the ‘risk of therapeutic inertia’ (i.e., the risk of doing nothing). Our definition expands current uses of the term ‘therapeutic inertia’ – focused on the failure to initiate or intensify therapy in a timely manner in a patient treatment context [84] – to capture its upstream determinants, including drug clinical research and its incorporation into evidence-based guidelines. The *risk* of therapeutic inertia is not simply regulatory conservatism; it includes a form of omission bias embedded within regulatory systems, which results from diffuse responsibility, procedural rigidity, and an under-recognition of the harms of inaction. COPCOV co-principal investigator Nicholas White has deemed this to be an *imposed* inertia and described it as ‘the construction of a complex of procedural obstacles and the relegation of common sense and experience…associated directly with reduced productivity’ (Interview 6). The risk of therapeutic inertia is, in this sense, hard to define and quantify. Yet, rough proxies exist, such as Whitney and Schreider’s ‘cost in lives’ methodology for estimating the negative consequences of ethics review delays [85].

COPCOV’s experience in an African country that did not obtain approval to recruit illustrates this risk. In this country, both the SOLIDARITY and COPCOV trials did not move forward because of what an investigator called the ‘the softer side of things, which you never hear of’, including the Ministry of Health’s reluctance to delegate authority to researchers. Sequential approval steps required investigators to ‘do the same thing three times’ through submission to a national and two local ECs, one of which refused to join AVAREF meetings and undertook a ‘kind of posturing…an attitude that we can do what we want, we are the solution’ (Interview 9). These challenges reflected not just regulatory risk aversion due to lingering safety concerns about HCQ, but also a fatal combination of strict procedural requirements and lack of appreciation of the value of clinical research.

## Conclusions

Returning to the metaphor of the epidemic curve of ambition, the risk of therapeutic inertia can be further conceptualised as the morbidity and mortality rendered by a pathogen (the delay in the use of effectual interventions, the use of ineffectual and harmful interventions) that could have been prevented if evidence for therapeutics was available to guide public health interventions. The risk of therapeutic inertia is neglected by the ICH-GCP system and sponsor risk management frameworks that underpin EC/NDRA bureaucratic formalism.

Judging the risk of medical interventions during a pandemic requires a comparator (i.e., the risk of trialling a particular medical intervention versus the risk of alternative courses of action, which includes doing nothing). Without this, stakeholders will naturally focus on reducing their risk in the narrowest sense, biasing towards deliberation and inaction. In the cases where risk is more broadly considered (using comparators or notions of ‘benefit’), the COPCOV experience underscores how this can still be highly subjective and can lead to esoteric decisions by ECs and NDRAs, even when they are well-resourced. Strong multilateral leadership can help to cut through approval bureaucracies by reducing subjectivity in interpreting clinical trial risks during health emergencies [20][23]. A global framework for emergency trial approval will be especially important for the response to future outbreaks like monkeypox, which may require sites across multiple countries to reach adequate power.

As London and Kimmelman wrote early in the COVID-19 pandemic, ‘Absent robust leadership from regulators, health authorities, and major funding bodies, the responsibility for coordinating research falls to a wide range of stakeholders who might normally pursue research on a more independent basis… the exigencies of crisis situations like global pandemics require exceptional steps to combine efforts, divide labour, and triage out low-value and duplicative research’ [86]. Without a recognition of these exceptional steps, trials like COPCOV will continue to succumb to the epidemic curve of ambition. Recognising the bureaucratic challenges facing clinical trials and the risk of therapeutic inertia is a first step for ‘triaging’ – or improving the efficiency, flexibility, and decision-making process quality of the clinical trial approval system, particularly for repurposed drugs or those with strong safety profiles. Otherwise, we will be in the same position in the next pandemic.

## Figures and Tables

**Fig. 6:**
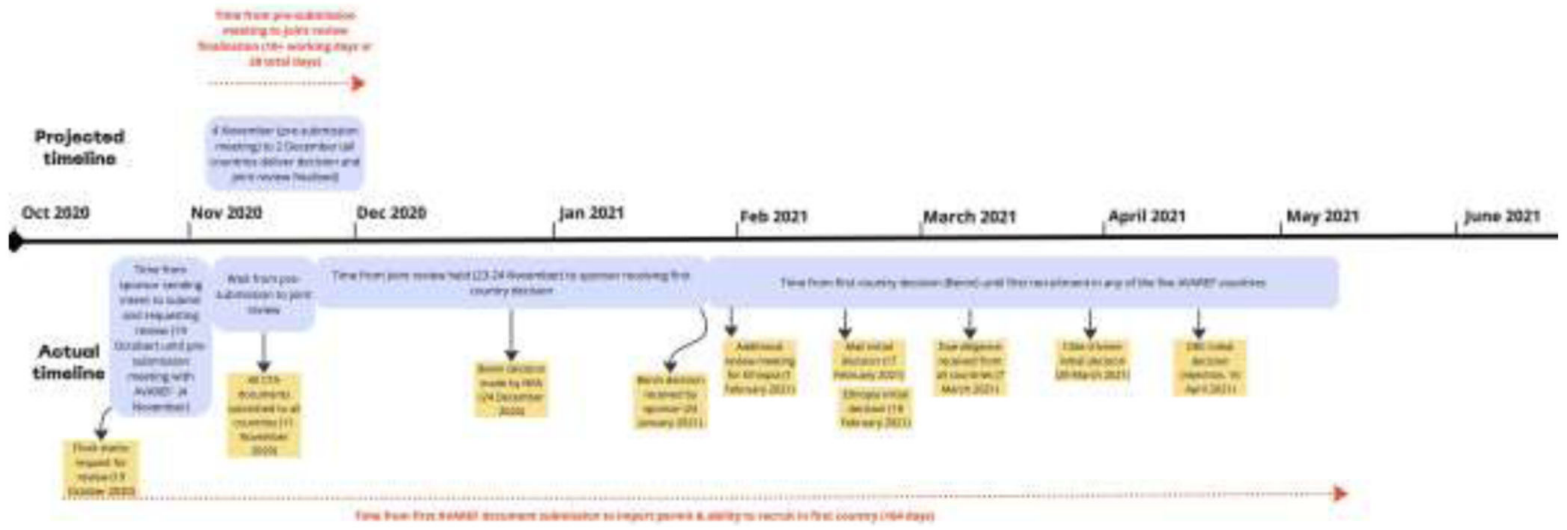
AVAREF’s emergency review timeline, as experienced by COPCOV

**Table 1:** Summary of findings and recommendations from major pre-COVID-19 studies on ethics committee and regulatory barriers for approval of clinical trials, with a focus on multi-country trials.

Study/Report	Findings and Recommendations
African Vaccine Regulatory Forum (AVAREF) Ebola vaccine acceleration evaluation (2008) [19]	Identified three major issues: (1) prolonged timelines for reviews of CTAs, (2) disparities in procedures and processes for reviews and CTA authorizations, and (3) unclear roles and responsibilities for ECs and NDRAs.
AIDS Clinical Trial Group (2014) [20]	Documented clinical trial “stumbling blocks”, including (1) EC approval challenges when long-term commitment to treatment seems unfeasible, (2) insurance unavailability for trial-related injuries, (3) lack of clear definition of EC authority and hierarchies, (4) challenges with biobanking, trade law, and emerging data/sample transfer agreements, and (5) unpredictability of regulatory frameworks.
WHO Guidance for mapping ethical issues in infectious disease outbreaks (2016) [21]	Chapter 8 (“Research during infectious disease outbreaks”) recommends that accelerated ethics review should take place in emergency situations, with one option being authorising advance review of generic protocols, with early discussion and collaboration with local ECs.
National Academies of Sciences, Engineering and Medicine report on Integrating Clinical Research into Epidemic Response: The Ebola Experience (2017) [22]	Concluded that RCTs are the most reliable way to identify relative risks and benefits of IMPs and every effort should be made to implement them during epidemics. Recommended that major research sponsors work with stakeholders in LMICs to build relationships between local ethics boards for surge capacity for ethics review (through writing MOUs on who will provide what services and how decisions will be made; establishing banks of experts in negotiation of clinical trial material transfer agreements and other essential components of collaboration for pro bono advice and support; developing templates for CTAs on data sharing, post-trial access, storage and analysis of biospecimens, and investments in local capacity). Highlighted two specific capacity-building initiatives: creating a Clinical Research Document Database (template for trials in low-resource settings) and an inter-epidemic research partnership structure for sharing clinical research know-how with LMICs.
WHO Africa Region report on clinical trial authorization and oversight in the AFRO region (2017) [23]	Pointed to inadequate preparedness for rapid initiation of RCTs during emergencies on the African continent; insufficient country ownership and financial resources for clinical trials; lack of EC and NDRA review and oversight harmonization (including multiple submission formats for CTA requirements and variable timelines); weak governance and lack of transparency (resulting in approvals required from multiple sources); limited biobanking capabilities for RCT specimens; and slow implementation of activities required to facilitate CTA review (including lack of full implementation of AVAREF meeting recommendations and poor benchmarking data). Recommended international and regional harmonization platform synchronisation.
CORTIFLU H1N1 trial (2012) [24]	Concluded that a multi-centre RCT of corticosteroids in intensive care unit patients with H1N1 pneumonia in France could have started a month earlier (hitting the peak flu wave) through parallel rather than sequential scientific, regulatory, financial, and ethics approvals and preparation of study drugs by local pharmacists.
West Africa Ebola, WHO Ethics Review analysis (2017) [25]	Recommended accelerating study approval during future public health emergencies through (1) consistent and complete submissions with information documents in appropriate languages, (2) close collaboration between local and international researchers from study inception, (3) generation of template data and sample sharing agreements to use during consultations on bio-banks, (4) formation of Joint Scientific Advisory and Data Safety Review Committees for all studies of specific intervention/group of interventions, (5) formation of Joint Ethics Review Committees with representatives of ECs of all institutions and countries, and (6) direct information exchange between chairs of advisory/safety review/research ethics committees.
Clinical Research Initiative for Global Health (2017) [26]	Cross-sectional survey of Research Bioethics project - based on the EU Clinical Trial Directive, ICH-GCP, WHO standards/operational guidance for ethics reviews, and 45 CFR (part 46) - identified ethics review systems and obstacles for interventional collaborative trials by non-profit and academic organizations. Found that the legal basis of ECs was mostly regulated for conflicts of interest but there was major variation in EC number nationally, accreditation, review timelines, transparency of decision, and training for committee members.
Bill & Melinda Gates Foundation “Disease X” working group (2018) [27]	Subject matter expert group determined that the global governance for facilitated regulatory pathways for a novel pathogen-induced pandemic generally lacked harmonization and transparency. High level overview identified 50 facilitated (‘agility promoting’) regulatory pathways internationally with the highest capabilities, focusing on either rapid assessment of countermeasures, rolling reviews, or conditional product authorisation (at the CTA and subsequent marketing levels).
PREPARE (2018) [28]	Survey of ECs and national competent authorities across European member states found that most respondents did not know of or did not have an expedited review process (and those that knew of a process were not aware of the standard operating procedures required to follow it).
ALERRT Ethics Preparedness, with WHO Global Health Ethics Team (2019) [29]	Workshop of EC members from across five continents convened in 2018 by WHO and the African Coalition for Epidemic Research (ALERRT) on facilitating ethics review during outbreaks highlighted the importance of definitive national approval before studies proceeded. Recommendations included: (1) developing national standard operating procedures for ECs for emergency response ethical review (which clarify terminology and expectations for pre-review of generic protocols), (2) strengthening communication procedures between national ECs and public health oversight bodies and authorities, (3) exploring mechanisms for multi-country emergency ethics consultation.
Nuffield Council on Bioethics (2020) [30]	Recommended that individual (not multi-country) national ECs/competent bodies prepare standard operating procedures for emergency review and supported voluntary harmonisation of criteria and procedures within this framework through ‘legitimate umbrella bodies’ like AVAREF.
Médecins sans Frontiéres Executive Review Board for Ebola (2017) [31]	MSF recommendations based on its ethics review board during Ebola: (1) establishing transparent EC communication mechanisms in advance, (2) setting-up joint pre-review or review mechanisms (including audio and video-conferencing mechanisms).

**Table 2:** COPCOV approval/decision times for the initial protocol and amendment to expand into community settings

Initial decision (ability to start a COPCOV protocol version for healthcare workers)	
Total bodies considering protocol	21 countries
	14 NRAs
	19 national or multi-site ECs
	22 local or institutional ECs/IRBs
Local EC/IRB decision (approval, rejection)	79 days (average)
	29 days (median, IQR 110 days)
	Range: 1-271 days
National or multi-site EC (approval, rejection)	76 days (average)
	55 days (median, IQR 96 days)
	Range: 15-193 days
Aggregate total days under EC consideration	2,759 days
	(1,321 days local ECs/IRBs, 1,430 national or multi-site)
NRA decision (approval, rejection)	89 days (average)
	91 days (median, IQR 74 days)
	Range: 4-206 days
Aggregate total days under NRA consideration	1,127 days
Days from first submission in a country to initial approval to recruit	109 days (average)
	104 days (median, IQR 85 days)
	Range: 29-248 days
**Secondary decision** **(ability to start COPCOV protocol version for community recruitment)**	
Total bodies considering protocol	11 countries
	8 NRAs
	8 national or multi-site ECs
	8 local or institutional ECs/IRBs
Local EC/IRB decision (approval, rejection)	57 days (average)
	50 days (median, IQR 39 days)
	Range: 7-90 days
National or multi-site EC (approval, rejection)	62 days (average)
	36 days (median, IQR 201 days)
	Range: 7-235 days
Aggregate total days under EC consideration	1,165 days
	(401 days local ECs/IRBs, 764 national or multi-site)
NRA decision (approval, rejection)	75 days (average)
	67 days (median, IQR 42 days)
	Range: 29-163 days
Aggregate total days under NRA consideration	599 days
Days from first submission in a country to initial approval to recruit	105 days (average)
	85 days (median, IQR 106 days)
	Range: 22-235 days

## Data Availability

The datasets generated and analysed during the current study are not publicly available, in compliance with GDPR and anonymisation as per the University of Oxford’s ethics clearance (CUREC) requirements. However, these data are available on reasonable request from the authors, subject to the removal of any personally identifying information, as are more detailed primary document bibliographies.

## List of abbreviations

AHC: academic health centre
ANTICOV: Platform Adaptive Trial for COVID-19 Outpatients in Africa
AVAREF: African Vaccines Regulatory Forum
COPCOV: Evaluation of Hydroxychloroquine or Chloroquine for the Prevention of COVID-19 Trial
COREQ: Consolidated Requirements for Qualitative Research
CQ: chloroquine
CRO: contract research organization
CTA: clinical trial agreement
CTU: clinical trials unit
CUREC: Oxford Central University Research Ethics Committee
DisCoVeRy: Trial of Treatments for COVID-19 in Hospitalised Adults
DSMB: Data Safety Monitoring Board
EC: ethics committee
ECG: electrocardiogram
EDCTP: European and Developing Country Clinical Trials Partnership
EMA: European Medicines Agency
EUA: emergency use authorization
EVD: Ebola virus disease
FDA: United States Food and Drug Administration
G7: Group of Seven
GloPID-R: Global Research Collaboration for Infectious Disease Preparedness
HCQ: hydroxychloroquine
HCW: health care workers
ICH: International Council for Harmonisation of Technical Requirements for Pharmaceuticals for Human Use
ICH-GCP: International Council on Harmonisation’s Good Clinical Practices
IMP: investigational medical product
IQR: interquartile range (of median)
IRB: institutional research board
KEMRI: Kenya Medical Research Institute
LMIC: low- and middle-income country
LOMWRU: Lao-Oxford-Mahosot Hospital Wellcome Trust Research Unit
MHRA: United Kingdom Medicines and Healthcare products Regulatory Agency
MORU: Mahidol Oxford Tropical Medicine Research Unit
MOU: memorandum of understanding
NASEM: United States National Institutes for Sciences, Engineering and Medicine
NDRA: national drug regulatory authority
NERVTAG: United Kingdom New and Emerging Respiratory Virus Threats Advisory Group
NIH: United States National Institutes of Health
NIHR: United Kingdom National Institute for Health and Care Research
NRA: national regulatory authority (alternative spelling for NDRA)
OECD: Organisation for Economic Co-operation and Development
OUCRU: Oxford University Clinical Research Unit (-Nepal, -Vietnam, -Indonesia)
OxTREC: Oxford Tropical Research Ethics committee
PAHO: Pan American Health Organization
R&D: research and development
RCT: randomised controlled trial
REC: research ethics committee
RECOVERY: Randomized Evaluation of COVID-19 Therapy Trial
REMAP-CAP: Randomized, Embedded, Multifactorial Adaptive Platform Trial for Community-Acquired Pneumonia
RhInnO: Council on Health Research for Development’s Research for Health and Innovation Organiser
SolidAct: European Union-RESPONSE Adaptive Platform Trial
SOLIDARITY: World Health Organization COVID-19 Therapeutics Trial
SRA: Stringent Regulatory Authority (now WHO-Listed Authority)
WHO: World Health Organization
UK: United Kingdom
ZAMRA: Zambia Medicines Regulatory Authority
