# Supplementary material for: Approval delays in multi-country COVID-19 trials: the case of COPCOV and the risk of therapeutic inertia
Source: Trials. 2025 Dec 16;27:58. doi: 10.1186/s13063-025-09300-z (PMC7618614; doi:10.1186/s13063-025-09300-z)

## **Supplement (Additional Files 1-6)**

**Additional File 1. Summary of interviews conducted with trial stakeholders.**

Trial stakeholders were identified in consultation with the principal investigators. A list of all site investigator teams was compiled for both prospective and recruiting sites, and JW systematically contacted all stakeholders requesting an interview, then performed snowballing to additional local investigators. Interviews were conducted on Teams and, in some cases, in-person in Indonesia, Thailand, and the United Kingdom. Background about the researcher (JW) was provided to all participants through a “one pager” about the project and its themes and an extensive participant information sheet. Semi-structured interviews were based on an interview guide with approximately fifteen general questions about the participants’ clinical trial background and experiences during COPCOV, including narrative questions about the participant’s background and case study questions. When permission was given to record audio, interviews were transcribed and stored according to the CUREC approval requirements at the University of Oxford (on OneDrive) and were analysed by JW.

| **Interview number** | **Stakeholder** | **Interview date(s)** |
| --- | --- | --- |
| 1 | University of Oxford insurance specialist | 29 March 2023 |
| 2 | AVAREF secretariat stakeholder | 6 September 2022, 10 November 2022 |
| 3 | AVAREF secretariat stakeholder | 6 September 2022 |
| 4 | AVAREF secretariat stakeholder | 6 September 2022, 25 October 2022, November 2022 |
| 5 | COPCOV co-principal investigator | Monthly sessions since February 2022 |
| 6 | COPCOV co-principal investigator | 26 April 2022, 10 May 2022, 25 August 2023, 5 September 2023 |
| 7 | COPCOV data safety management committee member | 10 June 2023, 13 June 2023 |
| 8 | COPCOV trial steering committee member | 20 May 2022 |
| 9 | African site investigator | 7 March 2023 |
| 10 | Former scientific director DNDi | 1 November 2022 |
| 11 | African prospective site investigator | 16 June 2023 |
| 12 | Good manufacturing practice (GMP) specialist | 21 November 2022 |
| 13 | Indonesia site administrator | 30 August 2023 |
| 14 | Indonesia site chief investigator | 1 September 2023, 2 September 2023 |
| 15 | Indonesia site coordinator | 29 August 2023 |
| 16 | Indonesia site coordinator | 7 September 2023 |
| 17 | Indonesia site coordinator and trial monitor | 27 April 2023, 1 September 2023, 2 September 2023 |
| 18 | Indonesia site data manager | 7 September 2023 |
| 19 | Indonesia site data manager | 7 September 2023 |
| 20 | Indonesia site investigator | 29 August 2023 |
| 21 | Indonesia site investigator | 4 September 2023 |
| 22 | Indonesia site laboratory manager | 30 August 2023 |
| 23 | Indonesia site nurse | 30 August 2023 |
| 24 | Indonesia site nurse | 7 September 2023 |
| 25 | Indonesia site pharmacist | 30 August 2023 |
| 26 | Indonesia site pharmacist | 7 September 2023 |
| 27 | Indonesia site study doctor | 4 September 2023 |
| 28 | Indonesia site study doctor | 4 September 2023 |
| 29 | Indonesia site study doctor | 30 August 2023 |
| 30 | Indonesia site study doctor | 30 August 2023 |
| 21 | Indonesia site study doctor | 7 September 2023 |
| 32 | Indonesia site study nurse | 30 August 2023 |
| 33 | Italy prospective site investigator | 13 April 2023 |
| 34 | Southeast Asia site investigator | 22 February 2023 |
| 35 | African site administrator | 3 May 2023 |
| 36 | African site coordinator | 3 May 2023 |
| 37 | African site lead investigator | 3 May 2023 |
| 28 | MORU, clinical trial support group representative | 27 May 2022 |
| 39 | MORU, administrator | 14 June 2022 |
| 40 | MORU, chief operating officer | 23 June 2022, 24 August 2023 |
| 41 | MORU, clinical trial support group representative | 5 June 2022 |
| 42 | MORU, clinical trials support group representative | July 2022, August 2023 |
| 43 | MORU, clinical trials support group representative | April 2022, May 2022 |
| 44 | MORU, data manager | 15 June 2022, 23 June 2022, 14 September 2023 |
| 45 | MORU, director | 23 June 2022 |
| 46 | MORU, financial coordinator | June 2022 |
| 47 | MORU, financial manager | 5 June 2022 |
| 48 | MORU, researcher | 15 June 2022 |
| 49 | MORU, shipping and logistics expert | 8 February 2023 |
| 50 | MORU, COPCOV monitoring lead | 22 June 2022 |
| 52 | MORU, COPCOV site coordinator | 29 April 2022, 23 June 2022, 25 August 2022, 8 September 2023 |
| 53 | MORU, COPCOV study coordinator | 24 August 2022, 26 September 2022, 10 March 2023 |
| 54 | MORU, trial statistician | 28 May 2022 |
| 55 | MORU, Thailand site coordinator | 22 June 2022 |
| 56 | South Asia site coordinator | 24 August 2023 |
| 57 | South Asia site lead investigator | 7 March 2023 |
| 58 | clinical trial insurance expert | 26 August 2023 |
| 59 | African site investigator | 21 February 2023 |
| 60 | Indonesia site lead investigator | 15 February 2023 |
| 61 | South Asia site lead investigator | 17 April 2023 |
| 62 | Prospective site investigator | 27 February 2023 |
| 63 | United Kingdom country lead investigator | 1 June 2022 |
| 64 | WHO Guideline Review Committee representative | 9 May 2023 |
| 65 | African site investigator | 2 March 2023 |

**Additional File 2. Semi-structured interview question guide (adapted based on stakeholder position and experience).**

1. When the COVID-19 pandemic began in early 2020, where were you based professionally?
   1. What is your academic/professional background and what was your role at the time?
2. What experience did you have (if any) with clinical trials before COVID-19?
3. How did you first come to be involved in the COPCOV trial? What was your role during the trial, and how did it evolve?
4. What COPCOV stakeholders (or local team members) did you work with during the trial?
5. Were you involved in other clinical trials during COVID-19? If so, in what capacity?
6. *If involved in site selection and recruitment projection* –
   1. How was your trial site selected?
   2. How many participants did you envision at the site(s), and did this change as healthcare workers received vaccines in January 2021? If so, in what ways?
   3. What timeline did you initially plan for trial recruitment?
7. *If involved in approvals process for the site(s) –*
   1. What did the approval process look like for the clinical trial application (protocol) in your country/at your trial site?
   2. Did you make more than one submission (e.g., for health care worker and then community participation)?
   3. What ethics and regulatory bodies were involved?
   4. What do you know about the mandates and composition of these bodies?
      1. Follow-up with information about national policies/documents available.
   5. Was there a process for expedited review of applications nationally or at your institution? If so, what was this process/track?
   6. Was the approval process sequential or parallel (give examples as needed if these terms are unclear)?
   7. Do you recall what the timeline was for approvals (e.g., from when you first submitted the application until you received an initial decision)?
      1. Follow-up with question about timeline for initial approval versus approval for other submissions, like for expanding from health care worker to community participation.
      2. If potential dates are known from Trial Master File, follow-up to ask if these seem correct. Follow-up about document availability if these were not available in the COPCOV Trial Master File.
   8. What comments, if any, did you receive from ethics committees, institutional research boards, and/or national regulatory authorities?
   9. How did communication work during the trial, including with the sponsor and regulatory/ethics authorities?
   10. How did COPCOV’s approval process and timeline compare to other trials you were involved in during COVID-19 or previous pandemics/health emergencies?
8. What worked well in terms of COPCOV site approval and/or activation?
   1. Were these specific to COPCOV? If not, what other experiences did you have with facilitators to clinical research, especially during health emergencies/pandemics?
9. What were the major challenges in terms of site approval and/or activation?
   1. Were these specific to COPCOV? If not, what other experiences did you have with barriers to clinical research, especially during health emergencies/pandemics?
10. *Depending on responses to #8-9 – Follow-up questions about risk calculations and politics at the site(s), e.g.:*
    1. How did clinical trial insurance work at your site/organisation?
    2. Were you aware of the ‘Surgisphere’ or Mehra et al./Lancet controversy in May 2020? If so, did this or other controversies around hydroxychloroquine have an impact on the COPCOV trial approvals, site activation, or recruitment processes? What was this impact?
    3. Were you aware of the World Health Organization’s guidelines on hydroxychloroquine as a therapeutic/prophylaxis (if not, explain the Living Guidelines and strong recommendation against the use of hydroxychloroquine)? Did this impact any part of your site/country’s approvals, site activation, or recruitment processes for COPCOV?
    4. Were there any other national or local guidelines around hydroxychloroquine that impacted your site?
    5. Are there documents or emails that you can share about the trial approval requirements, processes, timelines, or broader experiences?
11. *If involved in recruitment and site administration processes:*
    1. How was your site staffed? Do you know what the site budget was and whether this presented any challenges?
    2. When did recruitment begin? What was the final recruitment at the site? When was recruitment completed?
    3. How were trial supplies, including consumables and drugs, sourced? Did you encounter any challenges with obtaining supplies (including import permits, shipping, and labelling)? If so, what were they?
    4. What role did patient/public engagement play (if any) during COPCOV?
    5. Were any other organisations involved in your local recruitment/site administration (e.g., logistics companies, contract research organisations)?
    6. Are there any other issues you encountered or experiences that you want to share around COPCOV recruitment and the administration of your site(s)? (Examples: reimbursement, electronic data capture platform for symptoms, etc.)
12. *If involved with patient samples and/or site closure and data analysis processes:*
    1. What was your role with monitoring or patient samples (including analysis)?
    2. What issues were encountered during COPCOV in these areas?
13. Have you worked with contract research organisations (CROs) or site management organisations (SMOs) for clinical research in the past?
    1. Have you worked with any other for-profit bodies in the context of your clinical research?
    2. If so, in what capacity? What trial barriers/processes did they address?
14. Overall, what do you think worked and didn’t work in terms of the COPCOV trial approvals/governance processes?
    1. What governance or other reforms could have helped COPCOV or other clinical trials that you have been involved in, especially if they were multi-country trials or taking place during a health emergency?
    2. Any other thoughts about ethics/regulatory processes that presented bottlenecks and how they could be addressed?
15. Is there anything that we haven’t talked about that you would like to discuss, in terms of your experiences during COPCOV or with other COVID-19 clinical trials?
    1. Are there other people that I should talk with to better understand your site/country/organisation’s experiences with COVID-19 clinical research and the COPCOV trial?

**Additional File 3. Qualitative coding strategy.**

| **Initial inductive interview codes** | **Thematic domains used for analysis** |
| --- | --- |
| Regulatory reliance processes | Flexibility |
| Expedited or emergency review processes (availability) | Efficiency, flexibility |
| Expedited or emergency review processes (usage) | Efficiency |
| Regional regulatory or ethics harmonisation processes (availability) | Flexibility |
| Regional regulatory or ethics harmonisation processes (usage) | Efficiency |
| Number of documents required | Efficiency |
| Scope of documents required | Efficiency, decision-making coherence |
| Processes for notarising and submitting documents | Efficiency |
| Training of ethics or regulatory body members | Decision-making coherence |
| Funding or resources of ethics or regulatory bodies | Efficiency, decision-making coherence |
| Political or news influences (e.g., safety perceptions of drug) | Decision-making coherence |
| Dependency on external relationships (personal, professional networks) | Decision-making coherence |
| Feedback about local contexts | Decision-making coherence |
| Sequential approval processes | Efficiency |
| Upstream delays to clinical trial applications (e.g., insurance, budgeting) | Efficiency |
| Communication between sponsor and ethics/regulatory bodies | Efficiency, decision-making coherence |
| Communication between country ethics/regulatory bodies | Efficiency, decision-making coherence |
| Process for communicating decisions (approval/rejection) | Efficiency |
| Rationale given for approval/rejection | Decision-making coherence |
| Transparency of decision-making | Efficiency, decision-making coherence |
| Alignment of feedback between ethics/regulatory bodies | Decision-making coherence |
| Safety or efficacy concerns due to Mehra et al. (*Lancet* 2020) | Decision-making coherence |
| Citation of safety or efficacy concerns due to WHO guidance | Decision-making coherence |
| Ethics or regulatory bodies acting within or outside of mandates | Decision-making coherence |

**Reflexivity statement:** Our data is derived from the ‘COPCOV History Project’, a sub-project within the wider COVID-19 Accelerator Grant for the COPCOV trial. A single researcher (JW) was employed for this project, and permissions received from the central ethics body at the University of Oxford limited conducting of oral history interviews and viewing of interview transcripts to this researcher. The co-author (WS) is a principal investigator of the COPCOV trial and assisted with the development of the thematic analysis framework, based on JWs initial inductive coding. JW was embedded within the ongoing COPCOV trial from January 2022-December 2023 as a trial historian and was provided with access to many non-public trial records. She developed close working relationships with trial stakeholders and was given internal documents from this position of trust. JW conducted all interviews to promote objectivity (WS was involved in the trial from its origins in 2020 and JW began only after the trial was closing out in all countries). Our methods followed recommendations for analysis of oral history data (e.g., Firouzkouhi M, Zargham-Boroujeni A. Data Analysis in Oral History: A New Approach in Historical Research. Iranian Journal of Nursing and Midwifery Research. 2015;20(2):161-4.). As a native English speaker conducting interviews in English in Indonesia, Thailand, and other field settings, JW may not have fully captured the experiences of some local trial stakeholders. She sought diverse sources (e.g., interviews with multiple stakeholders from each country) to mitigate potential biases and backed-up stakeholder statements with emails and written documentation where possible. The authors acknowledge that JW’s analysis of the COPCOV trial is that of a ‘critical friend’, and that our choice to limit access of other researchers to the trial transcripts (as required under the University of Oxford CUREC) and to de-identify trial sites and investigators (as requested by many site investigators who have ongoing clinical research collaborations) increases the risk of bias in the qualitative analysis. This risk was reduced through using specific and manifest codes (second-level coding, based on input from WS about first-level codes), as well as by using broad, non-exclusive thematic domains (third-level coding). Reproducibility is also limited by the availability of primary sources, many of which are confidential. JW has saved copies of these source documents and can share them on reasonable request, subject to the removal of any personally identifying information.

**Additional File 4. Documents required for initial COPCOV approval by the Kenyan NDRA, the Pharmacy & Poisons Board (PPB).**

Sponsor (PI) letter requesting for the review and approval of the study

Decision letter from Kenya Centre Scientific Committee (CSC) secretary

Letter from PI with responses to CSC reviewers’ comments

Oxford Tropical Research Ethics Committee (OxTREC) sponsor letter

OxTREC approval of master protocol amendment v4.0

Master protocol (v4.0)

Master protocol (v5.0)

Site-Specific Addendum (v1.0)

Site-Specific Addendum (v2.0)

Participant information sheet (PIS)

Master English Informed Consent Form (ICF) v4.0)

Kenya English ICF (v1.0)

Kenya Kiswahili ICF (v1.0)

Consent translation, back translation certificate

Data collection forms

Master Case Report Form (CRF) Binder (v4.0)

Master ePRO (v4.0)

Master Participant ID card (v4.0)

Investigational Medical Product (IMP)

IMP Dossier test product (v2.0)

IMP Dossier placebo (v2.0)

Investigator’s Brochure (SmPC)

Recruitment materials – sensitization messages (WhatsApp/Email/Text message v1.0)

Recruitment materials – trial flyer (v1.0)

CVs of non-KEMRI investigators (7)

Investigator’s ethics certificates

Investigator’s current practicing licenses

Professional indemnity insurance – PI for KEMRI-CGMRC investigators

Professional indemnity insurance – PI for KEMRI-CGHR investigators

Professional indemnity insurance – PI for Aga Khan University Hospital investigators

Trial insurance – insurance quotation for University of Oxford

Trial insurance – University of Oxford premium invoice (Kenya sites)

Trial insurance – evidence of payment for insurance

Support letter from Coast General Teaching & Referral Hospital

Support letter from Jaramogi Oginga Odinga Teaching & Referral Hospital

Support letter from Mbagathi County Hospital and KWTRP agreement

Support letter from Aga Khan University Hospital

Declaration of financial disclosure/conflict of interest by PI

Declaration of financial disclosure/conflict of interest by applicant

Certificate of suitability (Stability data of the IMP supporting the intended shelf life)

GMP certificate of the IMP from the site of manufacture

GMP certificate (Accord – study drug manufacturer)

GMP certificate (Piramal – secondary packaging site)

Approved copies of labels

CVs for KEMRI and non-KEMRI investigators

GCP training certificates

Data Safety and Monitoring Board (DSMB) charter (v2.0)

Draft monitoring plan (v1.0) – final version to be sent later

Study budget

Statistical analysis plan (SAP, v1.0)

Study site capacity

Summary of previous supporting studies (v1.0), Document on why HCQ may work for prevention and not treatment, JAMA Review: Pharmacological Treatments for COVID-19

Prescribing guidelines for physicians enrolling and supervising participants in the COPCOV study (v1.0)

Proof of registration of the study with the Pan-African Clinical Trials Registry (PACTR)

Registration document of the clinical trial in the Kenya registry

Draft community engagement plan (v1.0)

Physician guidance on HCQ use (v1.0)

UK MHRA fall-out response documents: letter from MHRA allowing resumption of COPCOV trial, press release by MHRA about the green light to resume recruitment in the trial, position statement by COPCOV investigators (10th June 2020), Letter by COPCOV PIs on MHRA greenlight to resume trial, retraction statement by the Lancet

Site-specific addendum (v2.0)

PIS/ICF (v2.0, English and Kiswahili)

International Protocol (v5.0)

Approval letter by OxTREC for version 5.0 of international protocol

Copy of favourable opinion letter from KEMRI SERU (responses dated 23rd July 2020, 28th July 2020 and 2nd September 2020)

**Additional File 5. COPCOV’s United Kingdom Clinical Trial of Investigative Medical Products (CTIMP) approvals process and regulatory hurdles.**

**
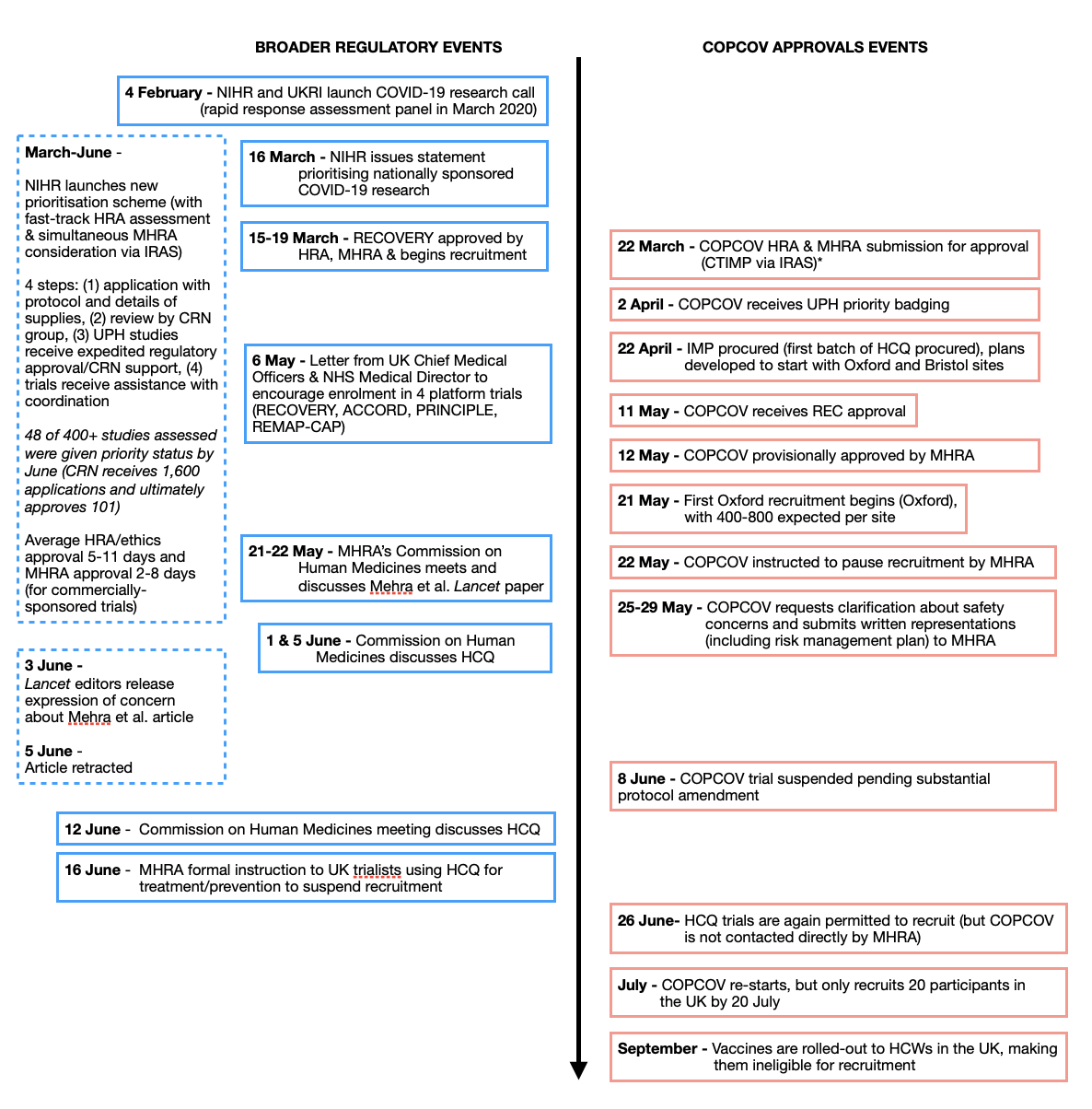
**

Broader regulatory approvals infrastructure and decisions are on the left (blue) and COPCOV submissions and approvals are on the right (orange). The Integrated Research Application System (IRAS) is designed to allow parallel consideration of a Clinical Trial of an Investigative Medical Product (CTIMP) trial protocol and required documents by a designated research ethics committee (REC) of the Health Research Authority (HRA) and the Medicines and Healthcare products Regulatory Agency (MHRA). The National Institute for Health Research (NIHR)’s Clinical Research Network (CRN) Support Unit Group performed Urgent Public Health (UPH) banding, which prioritised some trials that would test a vaccine/prophylactic/early treatment/diagnostic for COVID-19 and produce an interim or final published result within one year. Prioritised trials would receive preferential NIHR CRN support, thereby enhancing their ability to recruit using National Health System (NHS) resources. Compared to many countries, including Kenya (Additional File 4), the CTIMP/IRAS system required few documents in addition to the detailed COPCOV trial protocol (these included an IMP dossier, cover letter, and GMP certificate/manufacturer’s authorisation). After receiving UPH badging and final regulatory approval, the COPCOV trial began recruitment within a week and had 250 HCWs register interest its first day. However, on the first day of recruitment in Oxford, the MHRA’s body for addressing high-risk trials, the Commission for Human Medicines, deliberated the Mehra et al. paper pre-print. Trials using HCQ for a prophylaxis and treatment, including COPCOV, were instructed to pause recruitment. More documents were then required to obtain permission to re-start enrolment. These included, among others, a detailed risk analysis plan and Data Safety Monitoring Board minutes. Regulatory permission to re-start the trial was delayed for weeks after the retraction of the Mehra et al. paper, and the trial never regained momentum in the United Kingdom. UKRI is United Kingdom Research & Innovation.

**Additional File 6. Example of redacted meeting minutes obtained from the UK MHRA’s Commission on Human Medicines through a 2022 Freedom of Information request by the authors.**


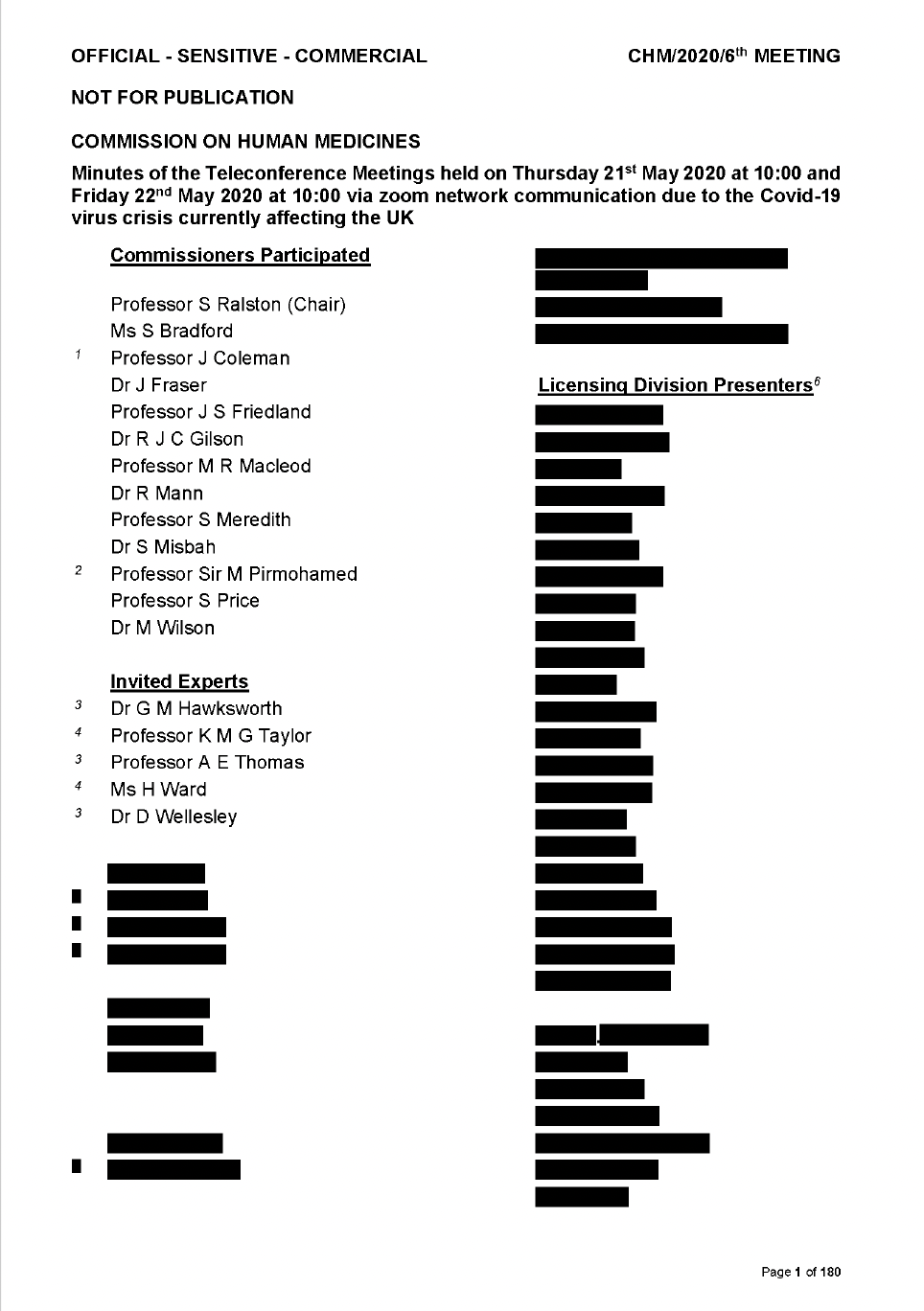

Supplement: Supplementary file 1 — Additional file 1. Summary of interviews conducted with trial stakeholders. Additional File 2. Semi-structured interview question guide (adapted based on stakeholder position and experience). Additional File 3. Qualitative coding strategy. Additional File 4. Documents required for initial COPCOV approval by the Kenyan NDRA, the Pharmacy & Poisons Board (PPB). Additional File 5. COPCOV’s United Kingdom Clinical Trial of Investigative Medical Products (CTIMP) approvals process and regulatory hurdles. Additional File 6. Example of redacted meeting minutes obtained from the UK MHRA’s Commission on Human Medicines through a 2022 Freedom of Information request by the authors. [file 13063_2025_9300_MOESM1_ESM.docx]
